# Severe undernutrition in children affects tuberculin skin test performance in Southern India

**DOI:** 10.1371/journal.pone.0250304

**Published:** 2021-07-16

**Authors:** Divya Reddy, Yicheng Ma, Subitha Lakshminarayanan, Swaroop Sahu, Laura F. White, Ayiraveetil Reshma, Gautam Roy, Padmini Salgame, Selby Knudsen, Chelsie Cintron, Jerrold J. Ellner, C. Robert Horsburgh, Sonali Sarkar, Natasha S. Hochberg

**Affiliations:** 1 Department of Medicine, Pulmonary Division, Albert Einstein College of Medicine, Bronx, New York, United States of America; 2 Department of Biostatistics, Boston University, School of Public Health, Boston, Massachusetts, United States of America; 3 Department of Preventive and Social Medicine, Jawaharlal Institute of Postgraduate Medical Education and Research, Puducherry, India; 4 Department of Medicine, Rutgers New Jersey Medical School, Newark, New Jersey, United States of America; 5 Department of Medicine, Section of Infectious Diseases, Boston University, School of Medicine, Boston, Massachusetts, United States of America; 6 Department of Epidemiology, Boston University, School of Public Health, Boston, Massachusetts, United States of America; The University of Georgia, UNITED STATES

## Abstract

**Background:**

Undernutrition impairs immunity to *Mycobacterium tuberculosis* and is a risk factor for tuberculosis disease (TB). We aim to investigate if severe undernutrition affects the tuberculin skin test (TST) response among household contacts (HHCs) of pulmonary TB cases.

**Methods:**

We analyzed data from HHCs (> five years) of pulmonary TB cases in Southern India. Undernutrition was defined as per World Health Organization based on body mass index (BMI) for adults (undernutrition 16–18.4 and severe undernutrition <16 kg/m^2^) and BMI relative to the mean for children (undernutrition 2SD-3SD and severe undernutrition < 3SDs below mean). Univariate and multivariate models of TST positivity (> five mm) were calculated using logistic regression with generalized estimating equations.

**Results:**

Among 1189 HHCs, 342 were children (age 5–17 years) and 847 were adults. Prevalence of TST positivity in well-nourished, undernourished and severely undernourished children was 135/251 (53.8%), 32/68 (47.1%), and 7/23 (30.4%) respectively; among adults, prevalence of TST positivity was 304/708 (42.9%), 43/112 (38.4%) and 12/26 (46.2%), respectively. Severe undernutrition in children was associated with decreased odds of TST positivity (adjusted odds ratio 0.3; 95%CI 0.1–0.9).

**Conclusion:**

Severe undernutrition in children was associated with decreased odds of TST positivity. False-negative TSTs may result from undernutrition; caution is warranted when interpreting negative results in undernourished populations.

## Introduction

Tuberculosis (TB), the leading infectious cause of death worldwide, affected 10 million people in 2017 [1]. Aerosolized droplets generated by an individual with active TB are the primary mode of spread. After inhaling such droplets, persons who become infected are largely able to control the infection and prevent progression to primary disease [2]. This *Mycobacterium tuberculosis (Mtb)* infection or “latent tuberculosis infection” is diagnosed by a positive tuberculin skin test (TST) or interferon gamma release assay (IGRA) in the absence of clinical signs or symptoms of disease [3]. In 2014, an estimated 1.7 billion individuals worldwide were infected with *Mtb* infection; South-East Asia, Western-Pacific, and Africa regions had the highest prevalence of infection and accounted for ~80% of those infected [4].

Several factors are linked to the progression of *Mtb* infection to disease, which occurs in 5–15% of infected individuals over their lifetime [5]. These factors include age less than five years, human immunodeficiency virus (HIV) infection, use of immunosuppressive medications such as glucocorticoids and tumor necrosis factor-α (TNF-α) inhibitors, chronic kidney disease, smoking, diabetes mellitus, and undernutrition [6]. Of these, undernutrition is of particular concern due to a high co-prevalence with *Mtb* infection in less-developed countries. According to the United Nations Food and Agricultural Organization (FAO), of the estimated 815 million people worldwide that are undernourished, the majority are from Asia (552 million, 68%) and sub-Saharan Africa (191 million, 23%) [7]. Additionally, Asia and Africa have high rates of undernutrition in children under five years of age which may compound the TB risk associated with young age [7].

The association of undernutrition with progression of *Mtb* infection to TB has been well established [8–11]. However, we recently reported in a meta-analysis that being underweight was not associated with a higher risk of a positive TST or IGRA [12]. The objective of this study, therefore, was to address whether severe undernutrition was associated with blunting of the TST and to identify other predictors of *Mtb* infection among household contacts (HHCs) of TB cases.

## Materials and methods

### Study setting and study design

This sub-study is part of an ongoing community-based observational HHC study, the Regional Prospective Observational Research for TB (RePORT) cohort, conducted by Jawaharlal Institute of Postgraduate Medical Education and Research (JIPMER) in collaboration with Boston Medical Center (BMC) and Rutgers New Jersey Medical School. Detailed descriptions of the study protocol and recruitment methods have previously been published [13, 14].

Briefly, pulmonary TB cases in Puducherry and Cuddalore and Villapuram districts of Tamil Nadu were identified and recruited from the National Tuberculosis Elimination Program (NTEP) district microscopy centers and primary healthcare centers. Index case inclusion criteria were: 1. New diagnosis of smear-positive culture-confirmed pulmonary TB. 2. Intake of less than three doses of anti-TB medication. 3. No history of TB or TB treatment. 4. Absence of multidrug resistant TB and/or contact with a multidrug resistant TB case. 5. Age greater than five years. Identification of TB cases through NTEP allowed for recruitment of a study sample representative of the TB cases in the community.

If TB cases reported at least two HHCS, these individuals were approached for participation. A HHC was defined as someone who on average slept under the same roof, shared at least one meal per day, or watched television (or the equivalent) with the TB case at least five days per week based on the groups previous work in Brazil [15–17]. Inclusion criteria for HHCs included: 1. Age > five years. 2. Had lived with the TB case ≥ three months prior to enrollment. 3. Was willing to get a TST. Exclusion criteria included: 1. Did not intend to live with the TB case in the following year or stay in the area for study duration. 2. Had a history of TB.

The main aim of the parent protocol (RePORT cohort) is to identify biomarkers of treatment failure among index TB cases and biomarkers of TB development among household contacts with the recruitment of smear positive index TB cases and their corresponding HHCs. Children less than six years of age were excluded in both the groups as the yield of smear positivity in children with TB is low and TST positive child HHCs are treated with Isoniazid preventive therapy as per standard of care. Written informed consent was obtained by all participants.

### Data collection

For TB cases, questionnaires were used to obtain demographic, household, clinical information including co-morbidity and medication data, and HIV testing was performed. Sputum was collected for concentrated acid-fast bacilli (AFB) smear and Löwenstein–Jensen and mycobacterial growth indicator tube (MGIT) cultures.

All HHCs were screened for active TB and enrolled within eight weeks of TB case enrollment. Information was collected on demographic and clinical characteristics as well as on exposure to the TB case. Bacillus Calmette-Gúerin (BCG) vaccination status was determined by the presence of a scar. Height and weight were measured to determine body mass index (BMI). A Mantoux TST (including Tubersol, Lederle, Arkray, and SPAN Diagnostics, India) was administered to HHCs as per NTEP guidelines. In brief, 5 TU of Purified Protein Derivative (Lederle, Tubersol, Arkray, and SPAN Diagnostics, India) was placed intradermally on the forearm. The diameter of induration was measured in millimeters using the "ball-point" technique by trained technicians. A pair of digital calipers was used to measure the induration, which were regularly serviced to reduce digit bias. TSTs were read between two to five days due to variant tuberculin reactivity and persistence of positive TSTs up to seven days after testing [18, 19].

Paper questionnaires were scanned and transferred to BMC with Verity TeleForm Information Capture System software V10.8 (Sunnyvale, CA, USA), and read into a Microsoft Access (Seattle, WA, USA) database. Data quality checks were performed; errors were evaluated and rectified by the study team in India. One TB patient was enrolled into the study at the age of five years. The Institutional Review Board was notified regarding this study protocol deviation and data of this patient was retained for analysis.

### Study definitions

A TST ≥ five mm was considered positive. The modified Alcohol Use Disorders Identification Test (AUDIT-C) was used to assess alcohol use (score ≥ four in males and ≥ three in females is considered “hazardous” alcohol use) [20]. In adults (≥ 18 years of age), BMI was categorized as severe undernutrition (< 16 kg/m^2^), undernutrition (16–18.4 kg/m^2^), and normal/overweight (≥ 18.5 kg/m^2^) [21]. In children (6–17 years), nutritional status was defined by standard deviations (SD) relative to the mean BMI as determined by the World Health Organization (WHO): Normal < 2SD above and below; undernutrition between 2SD to 3SD below mean; severe undernutrition as < 3SDs below mean [22]. Household location was categorized into large city (population > 100,000), small city (50,000–100,000), town (other urban area) and rural (countryside). The multidimentional poverty index (MPI), based on education, health and living standards, was calculated to categorize the households as poor and not poor [23]. Crowding was assessed based on number of individuals per room.

### Statistical analyses

We analyzed data from patients recruited into the RePORT cohort from May 2014—March 2018. Descriptive statistics were calculated using chi-square or Fischer’s exact tests for categorical and t-tests for continuous variables. Both univariate and multivariable analyses, with TST status as the outcome, were performed using logistic regression fit with generalized estimating equations (GEE) to account for household-level clustering effects. Separate models were constructed for adults and children. Undernutrition status and variables with *p*-values ≤ 0.2 in univariate analyses were included in each multivariable model. Diabetes mellitus (in adults) and history of BCG vaccination were retained in the models due to known associations with TST positivity [24, 25]. As nutritional status is a component of the MPI calculation, MPI was excluded from the multivariable model evaluating the impact of undernutrition on TST. Similarly, as undernutrition status correlated with the number of meals shared per day with the TB case, the latter was not included in the multivariable models. All data analyses were performed using SAS 9.4 (SAS Institute, Cary, NC).

This sub-study protocol was reviewed and approved by the BMC, Albert Einstein College of Medicine and Rutgers Institutional Review Boards and the JIPMER Ethics and Scientific Advisory Committees.

## Results

### Demographic characteristics of study population

Of the 1395 HHCs screened for study enrollment, 27 were ineligible and 76 did not consent. 103 HHCs of the remaining 1292 HHCs were excluded due to missing information. Our study therefore analyzed data from 1189 HHCs of 401 TB cases. Only 4/1189 (0.34%) HHCs were found to have active TB disease when screened at the time of study enrollment. The median age of all HHCs was 26 years (range: 5–93); 847 (71.2%) were adults and 342 (28.8%) children (Tables 1 and 2). Females accounted for 538/847 (63.5%) adults and 164/342 (48.0%) children. The largest proportion (520/1185 [43.9%]) of HHCs were children of TB cases, followed by siblings (n = 281 [23.7%]) of TB cases. Of adults, 112/847 (13.2%) were undernourished and 26/847 (3.1%) were severely undernourished. Among children, 68/342 (19.9%) and 23/342 (6.7%) were undernourished and severely undernourished, respectively. Tables 1 and 2 provide further details on demographic, household and index case characteristics in addition to details of time spent with index case by adult and child HHCs respectively.

**Table 1:** Univariate and multivariate models of predictors of tuberculin skin test positivity in adult (≥18 years of age) household contacts of pulmonary TB cases in India, n = 847.

Household Contact Characteristics	Total n = 847	Tuberculin skin test status		Univariate OR (95% CI)	p-value	Multivariate OR (95% CI)
		Positive n = 487	Negative n = 360			
		n (%)	n (%)			
*Demographic characteristics*						
Median age, years (range)	35 (18–93)	37 (18–80)	32.5 (18–93)	1.01 (1.00, 1.02)	0.03	1.0 (0.99–1.02)
Sex						
Male	309 (36.5%)	156 (32.0%)	153 (42.5%)	reference	0.0004	reference
Female	538 (63.5%)	331 (68.0%)	207 (57.5%)	1.6 (1.24–2.10)		1.4 (0.97–2.02)
Median years of education (range)	8 (0–19)	8 (0–18)	9 (0–19)	0.97 (0.95, 1.00)	0.03	1.0 (0.97–1.04)
Relationship to index case					0.005	
Spouse	263 (31.1%)	175 (35.9%)	91 (25.3%)	1.5 (1.05, 2.23)		1.3 (0.83–2.06)
Parent	121 (14.3%)	69 (14.2%)	52 (14.4%)	1.2 (0.72, 1.88)		1.1 (0.62–1.90)
Child	266 (31.4%)	134 (27.5%)	129 (35.8%)	0.8 (0.55, 1.21)		0.9 (0.58–1.40)
Sibling/other	197 (23.3%)	109 (22.4%)	88 (24.4%)	reference		reference
Tobacco smoking status						
Current/former	89 (10.5%)	48 (9.9%)	41 (11.4%)	0.9 (0.62–1.44)	0.77	
Never	758 (89.5%)	439 (90.1%)	319 (88.6%)	reference		
Hazardous Alcohol use						
Yes	53 (6.3%)	25 (5.1%)	28 (7.8%)	0.7 (0.39, 1.18)	0.17	0.9 (0.46–1.69)
No	794 (93.7%)	462 (94.9%)	332 (92.2%)	reference		reference
BCG vaccination status						
Yes	721 (85.1%)	407 (83.6%)	314 (87.2%)	0.8 (0.51, 1.22)	0.28	0.7 (0.42–1.08)
No/Don’t know	126 (14.9%)	80 (16.4%)	46 (12.8%)	reference		reference
*Comorbidities*						
History of Diabetes Mellitus						
Yes	50 (5.9%)	31 (6.4%)	19 (5.3%)	1.4 (0.80, 2.46)	0.23	1.6 (0.68–3.95)
No/Don’t know	797 (94.1%)	456 (93.6%)	341 (94.7%)	reference		reference
Nutritional status					0.58	
Severely malnourished	26 (3.1%)	14 (2.9%)	12 (3.3%)	0.9 (0.42, 1.98)		1.0 (0.46–2.37)
Malnourished	112 (13.2%)	69 (14.2%)	43 (12.0%)	1.2 (0.81, 1.81)		1.3 (0.86–2.08)
Well-nourished	708 (83.7%)	404 (83.0%)	304 (84.7%)	reference		reference
*Variables related to time spent with TB case*						
Meals shared with TB case per day					0.56	
None	166 (19.6%)	95 (19.5%)	71 (19.7%)	reference		
1	385 (45.5%)	219 (45.0%)	166 (46.1%)	1.1 (0.71, 1.56)		
2	171 (20.2%)	94 (19.3%)	77 (21.4%)	0.98 (0.62,1.53)		
3 or more	125 (14.8%)	79 (16.2%)	46 (12.8%)	1.3 (0.82, 2.21)		
Sleeping Proximity relative to TB Case					0.04	
Same room, same bed	63 (7.4%)	40 (8.2%)	23 (6.4%)	1.5 (0.90, 2.63)		1.3 (0.74–2.42)
Same room, different bed	316 (37.3%)	194 (39.8%)	122 (33.9%)	1.4 (1.03, 1.83)		1.3 (0.97–1.79)
Other ^Τ^	468 (55.3%)	253 (52.0%)	215 (59.7%)	reference		reference
Hours spent caring for TB case					0.008	
0	145 (17.1%)	87 (17.9%)	58 (16.1%)	reference		reference
<1 hour per day	316 (37.3%)	160 (32.9%)	156 (43.3%)	0.8 (0.51, 1.19)		0.7 (0.46–1.12)
1–6 hours per day	347 (41.0%)	212 (43.5%)	135 (37.5%)	1.2 (0.78, 1.76)		0.8 (0.48–1.22)
> 6 hours per day	39 (4.6%)	28 (5.8%)	11 (3.1%)	2.0 (0.92, 4.54)		1.3 (0.56–3.15)
*Household characteristics*						
Location of household					0.001	
Large city	41 (4.9%)	18 (3.8%)	23 (6.5%)	reference		reference
Small city	32 (3.8%)	17 (3.6%)	15 (4.2%)	1.9 (0.59–6.02)		1.7 (0.53–5.68)
Town	365 (43.8%)	242 (50.5%)	123 (34.7%)	2.7 (1.13–6.60)		2.5 (0.97–6.22)
Rural	396 (47.5%)	202 (42.2%)	194 (54.7%)	1.5 (0.60–3.53)		1.3 (0.50–3.24)
Multidimensional poverty index*						
Poor	545 (64.3%)	301 (61.8%)	244 (67.8%)	0.8 (0.57–1.11)	0.17	
Not poor	302 (35.7%)	186 (38.2%)	116 (32.2%)	reference		
Wood fuel use						
Yes	405 (47.8%)	224 (46.0%)	181 (50.3%)	0.9 (0.63, 1.18)	0.35	
No	442 (52.2%)	263 (54.0%)	179 (49.7%)	reference		
Number of persons per room (median, range)	1.0 (0.25–6.33)	1.0 (0.25–6.33)	1.0 (0.29–6.33)	1.0 (0.86–1.23)	0.78	
*Index case characteristics*						
Median age, years (range)	47.0 (14.0–77.0)	47.0 (14.0–77.0)	49 (14.0–77.0)	0.99 (0.98–1.00)	0.10	
Sex (missing = 1)						
Male	658 (77.8%)	373 (76.8%)	285 (79.2%)	reference	0.77	
Female	188 (22.2%)	113 (23.3%)	75 (20.8%)	0.9 (0.64–1.39)		
Tobacco smoking status						
Current/Former	416 (49.1%)	236 (48.5%)	180 (50.0%)	0.98 (0.72–1.35)	0.90	
Never	431 (50.9%)	251 (51.5%)	180 (50.0%)	reference		
Hazardous Alcohol use						
Yes	404 (47.7%)	230 (47.2%)	174 (48.3%)	1.0 (0.73–1.37)	0.99	
No	443 (52.3%)	257 (52.8%)	186 (51.7%)	reference		
HIV infection						
Yes	15 (1.8%)	4 (0.8%)	11 (3.1%)	0.3 (0.07–1.18)	0.14	
No	832 (98.2%)	483 (99.2%)	349 (96.9%)			
Median duration of illness, weeks (range), missing = 3	4 (1.0–16.0)	4 (1.0–12.0)	4 (1.0–16.0)	0.97 (0.88–1.06)	0.50	
Median TTP-MGIT ^Ŧ^, hours (range), missing = 19	804 (115.0–2814.0)	807 (115.0–2814.0)	720 (115.0–2814.0)	1.0 (0.99–1.00)	0.40	

* Multidimensional poverty index not included in the multivariable model as its calculation includes the nutritional status of the household contact

^Τ^ Others: same building, different room, different building that is part of the same household, other.

^Ŧ^ Time-to-Positive Mycobacterial Growth Indicator Tube

**Table 2:** Univariate and multivariate models of predictors of tuberculin skin test positivity in child (5–17 years of age) household contacts of pulmonary TB cases in India, n = 342.

Household Contact Characteristics	Total n = 342	Tuberculin skin test status		Univariate OR (95% CI)	p-value	Multivariate OR (95% CI)
		Positive n = 174	Negative n = 168			
		n (50.9%)	n (49.1%)			
*Demographic characteristics*						
Median age, years (range)	13 (5–17)	13 (5–17)	13 (6–17)	1.1 (0.98–1.14)	0.16	1.1 (0.85–1.41)
Sex						
Male	178 (52.1%)	98 (56.3%)	80 (47.6%)	reference	0.13	1.4 (0.94–2.11)
Female	164 (48.0%)	76 (43.7%)	88 (52.4%)	0.7 (0.50–1.10)		reference
Median years of education (range)	8 (1–13)	9 (1–13)	8 (1–13)	1.1 (0.98, 1.14)	0.18	1.0 (0.75–1.25)
Relationship to index case (missing = 4)						
Child	254 (75.1%)	128 (74.4%)	126 (75.9%)	0.95 (0.55, 1.62)	0.84	
Sibling/other	84 (24.9%)	44 (25.6%)	40 (24.1%)	reference		
Tobacco smoking status						
Current/Former	2 (0.6%)	1 (0.6%)	1 (0.06%)	NA	NA	
Never	340 (99.4%)	173 (99.4%)	167 (99.4%)	NA		
Hazardous alcohol use						
No	342 (100%)	174 (100%)	168 (100%)	NA	NA	
BCG vaccination status						
Yes	286 (83.6%)	149 (85.6%)	137 (81.6%)	1.5 (0.85, 2.71)	0.16	1.6 (0.87–2.93)
No/Don’t know	56 (16.4%)	25 (14.4%)	31 (18.5%)	reference		reference
*Comorbidities*						
History of Diabetes Mellitus						
Yes	1 (0.3%)	1 (0.6%)	0 (0.0%)	NA	NA	
No/Don’t know	341 (99.7%)	173 (99.4%)	168 (100%)	NA		
Nutritional status					0.07	
Severely malnourished	23 (6.7%)	7 (4.0%)	16 (9.5%)	0.7 (0.40, 1.17)		0.3 (0.12–0.85)
Malnourished	68 (19.9%)	32 (18.4%)	36 (21.4%)	0.7 (0.37, 1.22)		0.8 (0.45–1.34)
Well-nourished	251 (73.4%)	135 (77.6%)	116 (69.1%)	reference		reference
Hours spent with the TB case in the same house everyday					1.00	
< 6 hours per day	40 (11.7%)	19 (10.9%)	21 (12.5%)	reference		
6–12 hours per day	266 (77.8%)	136 (78.2%)	130 (77.4%)	0.99 (0.49, 1.98)		
>12 hours per day	36 (10.5%)	19 (10.9%)	17 (10.1%)	0.99 (0.39, 2.57)		
Meals shared with TB case per day every day*					0.007	
None	43 (12.6%)	28 (16.1%)	15 (8.9%)	reference		
1	175 (51.2%)	99 (56.9%)	76 (45.2%)	0.7 (0.34, 1.59)		
2	109 (31.9%)	40 (23.0%)	69 (41.1%)	0.3 (0.16, 0.75)		
3 or more	15 (4.4%)	7 (4.0%)	8 (4.8%)	0.5 (0.14, 1.72)		
Sleeping Proximity relative to TB Case					0.53	
Same room, same bed	35 (10.2%)	18 (10.3%)	17 (10.1%)	1.2 (0.57, 2.65)		
Same room, different bed	158 (46.2%)	86 (49.4%)	72 (42.9%)	1.3 (0.81, 2.18)		
Other ^Τ^	149 (43.6%)	70 (40.2%)	79 (47.0%)	reference		
*Household characteristics*						
Location of household (missing = 3)					0.42	
Large city	8 (2.4%)	1 (0.6%)	7 (4.2%)	reference		
Small city	17 (5.0%)	10 (5.8%)	7 (4.2%)	8.3 (0.75–91.22)		
Town	144 (42.5%)	76 (43.7%)	68 (41.2%)	6.6 (0.72–61.32)		
Rural	170 (50.1%)	87 (50.0%)	83 (50.3%)	6.3 (0.68–58.28)		
Multidimensional poverty index						
Poor	219 64.0%)	109 (62.6%)	110 (65.5%)	0.9 (0.54–1.43)	0.61	
Not poor	123 (36.0%)	65 (37.4%)	58 (34.5%)	reference		
Wood fuel use						
Yes	188 (55.0%)	95 (54.6%)	93 (55.4%)	0.95 (0.60, 1.52)	0.84	
No	154 (45.0%)	79 (45.4%)	75 (44.6%)	reference		
Number of persons per room (median, range)	1.3 (0.33–6.33)	1.3 (0.40–6.33)	1.0 (0.33–6.33)	1.1 (0.85–1.34)	0.59	
*Index case characteristics*						
Median age, years (range)	42.0 (14.0–75.0)	42.0 (15.0–75.0)	42.0 (14.0–73.0)	0.99 (0.969–1.006)	0.18	
Sex (missing = 1)						
Male	259 (76.0%)	130 (75.1%)	129 (76.8%)	reference	0.83	
Female	82 (24.1%)	43 (24.9%)	39 (23.2%)	0.9 (0.54–1.64)		
Tobacco smoking status						
Current/Former	177 (51.8%)	82 (47.1%)	95 (56.6%)	0.7 (0.43–1.10)	0.12	
Never	165 (48.3%)	92 (52.9%)	73 (43.5%)	reference		
Hazardous Alcohol use						
Yes	157 (45.9%)	73 (42.0%)	84 (50.0%)	0.7 (0.44–1.14)	0.15	
No	185 (54.1%)	101 (58.1%)	84 (50.0%)	reference		
HIV infection						
Yes	1 (0.3%)	0 (0.0%)	1 (0.6%)	NA	NA	
No	341 (99.7%)	174 (100%)	167 (99.4%)			
Median duration of illness, weeks (range), missing = 3	4.0 (1.0–16.0)	4.0 (1.0–12.0)	4.0 (1.0–16.0)	0.9 (0.81–1.02)	0.16	
Median TTP-MGIT ^Ŧ^, hours (range), missing = 6	800.0 (221.0–2814.0)	801.0 (221.0–2814.0)	722.0 (300.0–1920.0)	1.0 (0.99–1.00)	0.41	

* Not included in the multivariable model due to its association with nutritional status

^Τ^ Others: same building, different room, different building that is part of the same household, other.

^Ŧ^ Time-to-Positive Mycobacterial Growth Indicator Tube.

### Predictors of TST positivity among adult HHCs

**Fig 1:**
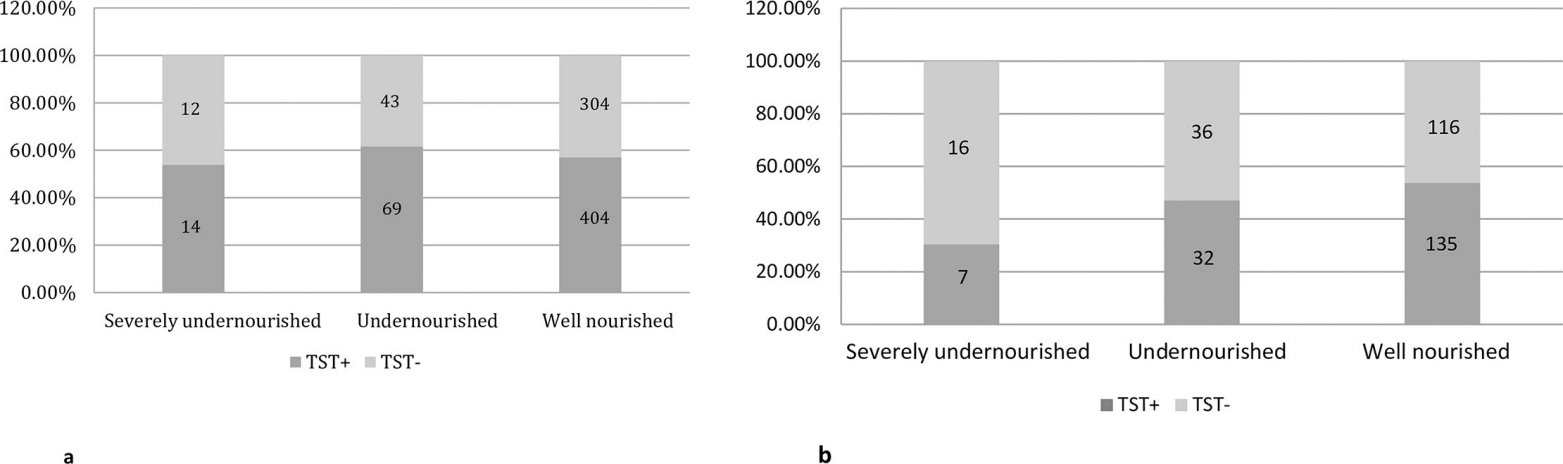
**a.** Distribution of tuberculin skin test positivity in adult household contacts of pulmonary TB cases, stratified by household contact nutritional status. **b.** Distribution of tuberculin skin test positivity in child (<18 years) household contacts of pulmonary TB cases, stratified by household contact nutritional status.

**Fig 2:**
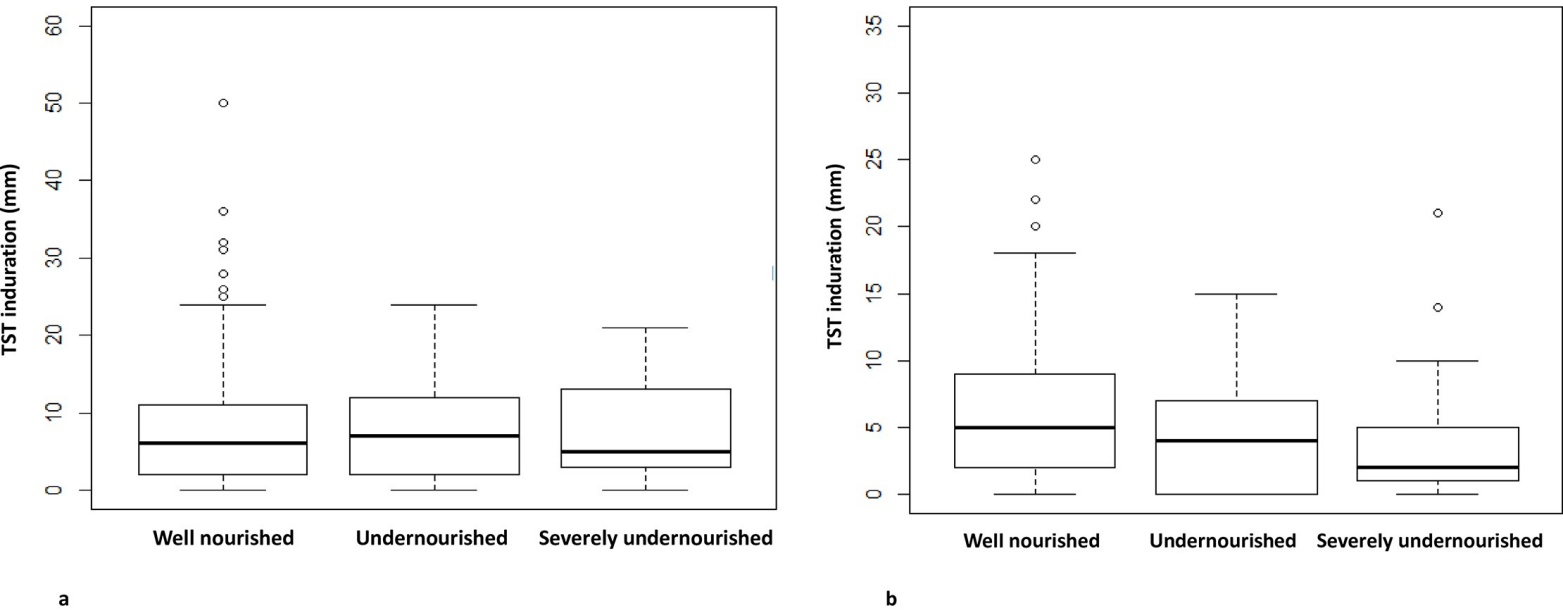
**a.** Tuberculin skin test induration size (mm) in adult household contacts of pulmonary TB cases, stratified by household contact nutritional status. *Well nourished vs undernourished adults HHCs (median TST xx mm vs yy mm; p = 0*.*31)*, *well nourished vs severely undernourished adult HHCs (median TST xx mm vs yy mm; p = 0*.*41) undernourished vs severely undernourished adult HHCs (median TST xx mm vs yy mm; p = 0*.*37)*. **b.** Tuberculin skin test induration size (mm) in child (<18 years) household contacts of pulmonary TB cases, stratified by household contact nutritional status. *Well nourished vs undernourished child HHCs (median TST 5 mm vs 2 mm; p = 0*.*31)*, *well nourished vs severely undernourished child HHCs (median TST 5 mm vs 2 mm; p = 0*.*03) undernourished vs severely undernourished child HHCs (median TST 4 mm vs 2 mm; p = 0*.*05)*.

Of 1189 HHC, 661 (55.6%) had a positive TST. Of the adults, 487/847 (57.5%) were TST positive. In univariate analyses, TST positive adults were more likely to be older (median 37 vs 32.5 years; p = 0.03), female (OR 1.6, 95%CI 1.24–2.10), the spouse of the TB case (OR 1.5, 95% CI 1.05–2.23), and have less education (median 8 vs 9 years; p = 0.03) compared to TST negative adult HHCs (Table 1). Sleeping in the same room but in a different bed was also associated with an increased odds of TST positivity (OR 1.4, 95% CI 1.03–1.83) compared to sleeping in a different room and/or different building, and TST positive adult HHCs were more likely (OR 2.7, 95%CI 1.13–6.60) to live in towns than large cities. As shown in Fig 1A, undernutrition status did not significantly differ (p = 0.58) between TST positive and TST negative adult HHCs. In a multivariable model, after adjusting for age, sex, years of education, relationship to TB case, BCG vaccination, hazardous alcohol use, diabetes mellitus, location of household, sleeping proximity to TB case and hours spent taking care of TB case, undernutrition (aOR 1.3, 95% CI 0.86–2.08) and severe undernutrition (aOR 1.0, 95% CI 0.46–2.37) were not significantly associated with TST positivity among adult HHCs. The median TST induration results didn’t significantly differ by nutrition status (p = 0.37) among adult HHCs (Fig 2A). We found that compared to large cities, living in a town was associated (aOR 2.5, 95% CI 0.97–6.22) with higher odds of TST positivity among adult HHCs; these results reached borderline significance.

### Predictors of TST positivity among child HHCs

Among child HHCs, 174/342 (50.9%) had positive TSTs. The prevalence of TST positivity in well-nourished, undernourished, and severely undernourished children decreased from 135/251 (53.8%), 32/68 (47.1%) to 7/23 (30.4%) respectively (Fig 1B). There was a significant decreasing trend (p = 0.02) in the proportion TST positive among child HHCs as nutritional status decreased from well nourished to severely undernourished. Among children, comorbidities and time spent with TB cases were not significantly associated with TST positivity in univariate analyses (Table 2). However, in a multivariable model after adjusting for age, sex, years of education and BCG vaccination, severe undernutrition in children was associated with a significantly lower odds (aOR 0.3, 95%CI 0.12–0.85) of TST positivity compared to well-nourished children, while undernutrition was not (aOR 0.8, 95% CI 0.45–1.34). The median ages of the child HHCs within the well nourished (13 years), undernourished (13 years) and severely undernourished (14 years) groups were not significantly different (p = 0.33). The median TST size in severely undernourished child HHCs (2 mm) was significantly smaller compared to the undernourished HHCs (4 mm; p = 0.05) and the well-nourished child HHCs (5 mm; p = 0.03; Fig 2B).

## Discussion

In a large cohort of household contacts (HHCs) of index pulmonary tuberculosis cases in South India, we found that ~55% of the HHCs had a positive TST; prevalence was high among adults (56%) and children (51%). Nearly one fifth of the studied HHCs were undernourished; among children, rates of undernutrition and severe undernutrition were high (20% and 7% respectively). We also found that severe undernutrition in child HHCs of pulmonary TB cases in India was associated with decreased odds of TST positivity.

Our finding of a lower odds of TST positivity in severely undernourished children suggests that the TST may not be a reliable measure of *Mtb* infection in severely undernourished children. Although there is no gold standard for diagnosis, we suspect that undernutrition led to a blunted immune response and under-diagnosis, rather than the true prevalence of infection being lower. Previous studies have been conflicting. In a study of 212 adults in Peru, protein energy undernutrition was associated with a lower likelihood of TST positivity [26], and among 6,608 adolescents in India, recent weight loss was associated with TST non-response [27]. Similarly, TST positivity post BCG vaccination increased with improved nutritional status and weight for age [28]. Other studies have failed to demonstrate an association [12, 29, 30]; however, all of these studies failed to account for the severity of undernutrition and/or rates of severe undernutrition were not documented. This is important as severe protein undernutrition in children (kwashiorkor) affected TST conversion six months post BCG vaccination while milder forms of undernutrition did not [31]. Furthermore, a retrospective evaluation of baseline TST results in patients who later developed active TB also showed that negative TSTs in malnourished individuals are false negatives [32].

The potential biologic mechanism for blunting of the TST in severe undernutrition is suppression of innate and adaptive immune responses [11, 33]. The TST relies on an effective delayed type hypersensitivity response; upon intradermal injection of Mtb antigens, pro-inflammatory cytokines are released that stimulate adhesion molecules to attract monocytes and T cells [34]. Protein-deprived *Mtb* H37Rv vaccinated mice and guinea pigs have shown diminished lymphoproliferation and defective T cell interaction with macrophages after purified protein derivative (PPD) stimulation [35]. In these models, *Mtb* antigen stimulation was associated with reduced interleukin-2, interferon-γ (IFN-γ) and tumor necrosis factor-α (TNF-α) and increased regulatory cytokines such as transforming growth factor-β (TGF-β) [36, 37].

Interferon gamma release assays (IGRAs) such as Quantiferon Gold In-Tube (QGIT) measure T cell IFN-ϒ release following stimulation with *Mtb* antigens and phytohemagglutinin-P (a non-specific T-cell stimulator) and rely on an adequate cellular response. Reports suggest that IGRA may also not be a reliable test in the setting of undernutrition, with an increase in indeterminate results and more negative tests compared with normal weight persons [12, 38].

The strength of the study lay in collection of detailed information using validated questionnaires and clinical measures for factors associated with *Mtb* infection risk (e.g., AUDIT-C, BMI). These data allowed us to perform multivariable analyses to control for potential confounders of the effect. One limitation was our inability to assess the effect on children less than six years old; these were excluded from the RePORT cohort. It is likely that severe undernutrition affects TST sensitivity in that group as well. The HIV status of our HHCs was unknown, but as Puducherry and Tamil Nadu have low HIV prevalence and rates are <1% in RePORT TB cases [14, 39], this is unlikely to be a significant risk factor for *Mtb* infection. The different formulations of purified protein derivative used for TSTs may have affected diagnosis of *Mtb* infection; however, as the prevalence of *Mtb* infection (55.6%) is comparable to other published studies, marked under-diagnosis is unlikely [40]. Median years of education among child HHCs likely is collinear with age and not a potential marker of community TB exposure like in the adults. We did not collect additional details on risk of community TB exposure in children as part of our study protocol. We analyzed the data to assess differences in time spent with the index TB case to explain the TST status. However, due to our small sample size of undernourished children [91/342 (26.6%)] we were unable to further explore differences in time spent with the index TB case by nutritional status. Lastly because we relied on reported history of diabetes mellitus, under-diagnosis is possible.

## Conclusion

As the number of TB cases decline, targeted screening and treatment of *Mtb* infected persons in high burden countries will be of increasing importance for TB eradication. As part of the stop TB strategy, the WHO currently recommends isoniazid preventive therapy (IPT) for recent TST converters, TST or IGRA positive children with close contact with a TB disease patient and TST positive persons living with HIV after TB disease has been excluded [41]. Our study found that severe undernutrition blunts the TST response in children and suggests that caution is warranted when interpreting TST results in this population. Due to the high prevalence of undernutrition in India and other TB-endemic countries [7], this study has public health relevance. TB programs might consider a lower cut-off point for severely undernourished young HHCs or presumptive treatment of *Mtb* infection regardless of TST status.

## References

[1] World Health Organization (WHO). Global Tuberculosis Report 2018. Geneva: WHO; 2018. https://www.who.int/tb/publications/global_report/tb18_ExecSum_web_4Oct18.pdf?ua = 1. Last accessed 1-12-2019

[2] Salgame P, Geadas C, Collins L, Jones-Lopez E, Ellner JJ. Latent tuberculosis infection—Revisiting and revising concepts. Tuberculosis. 2015 Jul;95(4):373–84. doi: 10.1016/j.tube.2015.04.003 26038289

[3] Getahun H, Matteelli A, Chaisson RE, Raviglione M. Latent Mycobacterium tuberculosis infection. N Engl J Med. 2015 May;372(22):2127–35. doi: 10.1056/NEJMra1405427 26017823

[4] Houben RMGJ Dodd PJ. The Global Burden of Latent Tuberculosis Infection: A Re-estimation Using Mathematical Modelling. PLoS Med. 2018 Apr;3(10): e1002152. 10.1371/journal.pmed.1002152 PMC5079585 27780211

[5] Shea KM, Kammerer JS, Winston CA, Navin TR, Horsburgh CR. Estimated Rate of Reactivation of Latent Tuberculosis Infection in the United States, Overall and by Population Subgroup. Am J Epidemiol. 2013 Dec 26;179(2):216–25. doi: 10.1093/aje/kwt246 24142915 PMC5547435

[6] Horsburgh CR Jr., Rubin EJ. Latent Tuberculosis Infection in the United States. N Engl J Med. 2011 Apr 14;364(15):1441–8. doi: 10.1056/NEJMcp1005750 21488766

[7] FAO. The State of Food Security and Nutrition in the World 2017. 2017 Sep 5;1–132. http://www.fao.org/home/en/. Last accessed 9-12-2018

[8] Edwards LB, Livesay VT, Acquaviva FA, Palmer CE. Height, Weight, Tuberculous Infection, and Tuberculous Disease. Archives of Environmental Health: An International Journal. 1971 Jan;22(1):106–12. 10.1080/00039896.1971.10665820 4992917

[9] Tverdal A. Body mass index and incidence of tuberculosis. Eur J Respir Dis. 1986 Nov;69(5):355–62. 3792471

[10] Lonnroth K, Williams BG, Cegielski P, Dye C. A consistent log-linear relationship between tuberculosis incidence and body mass index. International Journal of Epidemiology. 2010 Feb 6;39(1):149–55. doi: 10.1093/ije/dyp308 19820104

[11] Sinha P, Davis J, Saag L, Wanke C, Salgame P, Mesick J, et al. Undernutrition and Tuberculosis: Public Health Implications. J Infect Dis. 2019 Apr 16;219(9):1356–1363 doi: 10.1093/infdis/jiy675 30476125 PMC6941617

[12] Saag LA, LaValley MP, Hochberg NS, Cegielski JP, Pleskunas JA, Linas BP, et al. Low body mass index and latent tuberculous infection: a systematic review and meta-analysis. int j tuberc lung dis. 2018 Apr 1;22(4):358–65. doi: 10.5588/ijtld.17.0558 29562981

[13] Hochberg NS, Sarkar S, Horsburgh CR, Knudsen S, Pleskunas J, Sahu S, et al. Comorbidities in pulmonary tuberculosis cases in Puducherry and Tamil Nadu, India: Opportunities for intervention. PLoS ONE. 2017 Aug 23;12(8):e0183195. doi: 10.1371/journal.pone.0183195 28832615 PMC5568341

[14] Van Ness SE, Chandra A, Sarkar S, Pleskunas J, Ellner JJ, Roy G, et al. Predictors of delayed care seeking for tuberculosis in southern India: an observational study. BMC Infectious Diseases. 2017 Aug 15; 17(1):567– doi: 10.1186/s12879-017-2629-9 28806911 PMC5557420

[15] Jones-López EC, Acuña-Villaorduña C, Fregona G, Marques-Rodrigues P, White LF, Hadad DJ, et al. Incident Mycobacterium tuberculosis infection in household contacts of infectious tuberculosis patients in Brazil. BMC Infect Dis. 2017 Aug 18; 17(1): 576. doi: 10.1186/s12879-017-2675-3 28821234 PMC5563014

[16] Carlos Acuña-Villaorduña Edward C. Jones-López, Fregona Geisa, Patricia Marques-Rodrigues Mary Gaeddert, Geadas Carolina, et al. Intensity of exposure to pulmonary tuberculosis determines risk of tuberculosis infection and disease. European Respiratory Journal 2018 Jan, 51 (1) 1701578 doi: 10.1183/13993003.01578-2017 29348181 PMC6719538

[17] Acuña-Villaorduña C, Schmidt-Castellani LG, Marques-Rodrigues P, White LF, Hadad DJ, Gaeddert M, et al. Cough-aerosol cultures of *Mycobacterium tuberculosis* in the prediction of outcomes after exposure. A household contact study in Brazil. PLoS ONE 2018 October 29, 13(10): e0206384. doi: 10.1371/journal.pone.0206384 30372480 PMC6205616

[18] John M. Robertson, Dawn S. Burtt, Kay L. Edmonds, et al. Delayed Tuberculin Reactivity in Persons of Indochinese Origin: Implications for Preventive Therapy. Ann Intern Med.1996;124:779–784. doi: 10.7326/0003-4819-124-9-199605010-00001 8610946

[19] Centers for Disease Control and Prevention (CDC). Core Curriculum on Tuberculosis. Chapter 4. Testing for TB Disease and TB Infection. 2000 https://www.heartlandntbc.org/assets/training/mini-fellowship/PediatricToolBox/CDC/ed_training/publications/corecurr/Chap4/Chapter_4_Skin_Testing.htm. Last accessed 1-31-2021

[20] Reinert DF, Allen JP. The Alcohol Use Disorders Identification Test (AUDIT): A Review of Recent Research. Alcoholism: Clinical and Experimental Research. Blackwell Publishing Ltd; 2002;26(2):272–9. 11964568

[21] World Health Organization (WHO) Technical Report Series. Physical Status: The use and interpretation of anthropometry. Geneva: WHO; 1995. Jan 3;1–463. http://www.who.int/childgrowth/publications/physical_status/en/. Last accessed 9-09-2018. 8594834

[22] WHO Global Database on Child Growth and Malnutrition. 2003 May 2; 1–74. https://www.who.int/nutgrowthdb/en/. Last accessed 09-15-2018.

[23] Alkire S. and Santos M. E., “Measuring Acute Poverty in the Developing World: Robustness and Scope of the Multidimensional Poverty Index,” *World Development*, 52, 71–91, 2014.

[24] Cohn DL. The effect of BCG vaccination on tuberculin skin testing. Does it matter? Am J Respir Crit Care Med. 2001 Sep;164(6):915–6. doi: 10.1164/ajrccm.164.6.2107090c 11587970

[25] Lee M-R, Huang Y-P, Kuo Y-T, Luo C-H, Shih Y-J, Shu C-C, et al. Diabetes Mellitus and Latent Tuberculosis Infection: A Systemic Review and Metaanalysis. Clin Infect Dis. 2017 Mar;64(6):719–27. doi: 10.1093/cid/ciw836 27986673 PMC5399944

[26] Pelly TF, Santillan CF, Gilman RH, Cabrera LZ, Garcia E, Vidal C, et al. Tuberculosis skin testing, anergy and protein malnutrition in Peru. int j tuberc lung dis. 2005 Sep;9(9):977–84. 16161252 PMC2912519

[27] Uppada DR, Selvam S, Jesuraj N, Bennett S, Verver S, Grewal HM, et al. The tuberculin skin test in school going adolescents in South India: associations of socio-demographic and clinical characteristics with TST positivity and non-response. BMC Infectious Diseases 2014 14:1. 2014;14(1):571. doi: 10.1186/s12879-014-0571-7 25927335 PMC4243729

[28] Kielmann AA, Uberoi IS, Chandra RK, Mehra VL. The effect of nutritional status on immune capacity and immune responses in preschool children in a rural community in India. Bull World Health Organ. 1976;54(5):477–83. 1088398 PMC2366483

[29] Margolis B, Al-Darraji HAA, Wickersham JA, Kamarulzaman A, Altice FL. Prevalence of tuberculosis symptoms and latent tuberculous infection among prisoners in northeastern Malaysia. int j tuberc lung dis. 2013 Dec;17(12):1538–44. doi: 10.5588/ijtld.13.0193 24200265 PMC3913085

[30] Adetifa IMO, Muhammad AK, Jeffries D, Donkor S, Borgdorff MW, Corrah T, et al. A Tuberculin Skin Test Survey and the Annual Risk of Mycobacterium tuberculosis Infection in Gambian School Children. PLoS ONE. 2015 Oct 14;10(10):e0139354. doi: 10.1371/journal.pone.0139354 26465745 PMC4605652

[31] Satyanarayana K, Bhaskaram P, Seshu VC, Reddy V. Influence of nutrition on postvaccinial tuberculin sensitivity. Am J Clin Nutr. 1980 Nov;33(11):2334–7. doi: 10.1093/ajcn/33.11.2334 6776793

[32] Chan-Yeung M, Dai DL, Cheung AH, Felix HW Chan, Kai-Man Kam, Cheuk-Ming Tam, et al. Tuberculin skin test reaction and body mass index in old age home residents in Hong Kong. *J Am Geriatr Soc*. 2007;55(10):1592–1597. doi: 10.1111/j.1532-5415.2007.01316.x 17908061

[33] Rytter MJH, Kolte L, Briend A, Friis H, Christensen VB. The immune system in children with malnutrition—a systematic review. PLoS ONE. 2014;9(8):e105017. doi: 10.1371/journal.pone.0105017 25153531 PMC4143239

[34] Poulter LW, Seymour GJ, Duke O, Janossy G, Panayi G. Immunohistological analysis of delayed-type hypersensitivity in man. *Cell Immunol*. 1982;74(2):358–369. doi: 10.1016/0008-8749(82)90036-3 6762253

[35] Dai G, McMurray DN. Altered Cytokine Production and Impaired Antimycobacterial Immunity in Protein-Malnourished Guinea Pigs. *Infection and Immunity*. 1998;66(8):3562–3568. doi: 10.1128/IAI.66.8.3562-3568.1998 9673234 PMC108387

[36] Cegielski JP, McMurray DN. The relationship between malnutrition and tuberculosis: evidence from studies in humans and experimental animals. int j tuberc lung dis. 2004 Mar;8(3):286–98. 15139466

[37] Reynolds JV, Redmond HP, Ueno N, Steigman C, Ziegler MM, Daly JM, et al. Impairment of macrophage activation and granuloma formation by protein deprivation in mice. Cell Immunol. 1992;139(2):493–504. doi: 10.1016/0008-8749(92)90088-7 1310262

[38] Thomas TA, Mondal D, Noor Z, Liu L, Alam M, Haque R, et al. Malnutrition and helminth infection affect performance of an interferon gamma-release assay. Pediatrics. 2010 Dec;126(6):e1522–9. doi: 10.1542/peds.2010-0885 21059723 PMC3403682

[39] Open Government Data (OGD) Platform India. HIV Sentinel Surveillance. 2017 Dec 21;1–94. https://data.gov.in/keywords/hiv-sentinel-surveillance. Last accessed 09-15-2018.

[40] Fox GJ, Barry SE, Britton WJ, Marks GB. Contact investigation for tuberculosis: a systematic review and meta-analysis. European Respiratory Journal. 2012 Dec 31;41(1):140–56. doi: 10.1183/09031936.00070812 22936710 PMC3533588

[41] WHO End TB Strategy: Global strategy and targets for tuberculosis prevention, care and control after 2015. 2015. https://www.who.int/tb/post2015_strategy/en/. Last accessed 01-15-2021.

